# A recursive framework for predicting the time-course of drug sensitivity

**DOI:** 10.1038/s41598-020-74725-2

**Published:** 2020-10-19

**Authors:** Cheng Qian, Amin Emad, Nicholas D. Sidiropoulos

**Affiliations:** 1 Department of Electrical and Computer Engineering, University of Virginia, Charlottesville, VA USA; 2 Department of Electrical and Computer Engineering, McGill University, Montreal, QC Canada

**Keywords:** Cancer genomics, Computational models, Data integration

## Abstract

The biological processes involved in a drug’s mechanisms of action are oftentimes dynamic, complex and difficult to discern. Time-course gene expression data is a rich source of information that can be used to unravel these complex processes, identify biomarkers of drug sensitivity and predict the response to a drug. However, the majority of previous work has not fully utilized this temporal dimension. In these studies, the gene expression data is either considered at one time-point (before the administration of the drug) or two time-points (before and after the administration of the drug). This is clearly inadequate in modeling dynamic gene–drug interactions, especially for applications such as long-term drug therapy. In this work, we present a novel REcursive Prediction (REP) framework for drug response prediction by taking advantage of time-course gene expression data. Our goal is to predict drug response values at every stage of a long-term treatment, given the expression levels of genes collected in the previous time-points. To this end, REP employs a built-in recursive structure that exploits the intrinsic time-course nature of the data and integrates past values of drug responses for subsequent predictions. It also incorporates tensor completion that can not only alleviate the impact of noise and missing data, but also predict unseen gene expression levels (GEXs). These advantages enable REP to estimate drug response at any stage of a given treatment from some GEXs measured in the beginning of the treatment. Extensive experiments on two datasets corresponding to multiple sclerosis patients treated with interferon are included to showcase the effectiveness of REP.

## Introduction

Prediction of drug response based on patients’ clinical and molecular features is a major challenge in personalized medicine. A computational model that can make accurate predictions can be used to identify the best course of treatment for patients, or to identify therapeutic targets that can overcome drug resistance^1^. Considerable efforts have been made to identify molecular biomarkers of drug sensitivity and to develop computational models to predict drug response based on these biomarkers. Gene expression data is one of the most commonly used molecular data type in these studies, due to their high predictive ability, and numerous methods have been proposed for drug response prediction based on gene expression data^1–9^. However, many existing methods only use basal gene expression data (i.e., gene expression values before administration of the drug) and hence can only capture the influence of the steady state of the cells on their response to a drug. For example, Costello et al.^2^ analyzed 44 drug response prediction methods that employed gene expression profiles of breast cancer cell lines taken before treatment to predict dose-response values, e.g., GI50—the concentration for 50% of maximal inhibition of cell proliferation from a single time-point. In practice, however, for many diseases (e.g. cancers) the response to a drug changes over time due to various reasons such as the development of drug resistance or changes in the progress of the disease. To capture such changes at a molecular level, a collection of temporal gene expression profiles of samples over a series of time-points during the course of a biological process is necessary to provide more insights than a single (or two) time-point(s)^10^. Therefore, developing algorithms that can predict the drug response over time using time-course gene data is of great interest.

With the advancement of gene sequencing technologies, collecting gene expression levels (GEXs) over multiple time-points and their matched drug response values is now feasible. In parallel with these technological developments, there has been growing interest in the application of machine learning methods to analyze the time-course gene expression data. For example, time-course gene expression data can be used to not only identify longitudinal phenotypic markers^11,12^, but also assess the association between the time course molecular data and cytokine production in this HIV trial^13^ and predict drug response during a treatment^8,14^. In^15^, the authors proposed an integrated Bayesian inference system to select genes for drug response classification from time-course gene expression data. However, the method only uses the data from the first time-point, and hence does not benefit from the additional temporal information. Lin et al.^14^ presented a Hidden Markov model (HMM)-based classifier, in which the HMM had fewer states than time points to align different patient response rates. This discriminative HMM classifier enabled distinguishing between good/bad responders. Nevertheless, choosing the number of states for this HMM is a major practical issue. In addition, this method cannot handle missing data and it requires the full knowledge of GEXs at all time-points a priori. This implies that the HMM may not be able to predict drug response at multiple stages in future time points, since the corresponding GEXs are not measurable.

The time-course gene expression data contains the GEXs of different patients over a series of time points, which can be indexed as patient-gene-time and represented as a three-dimensional tensor. Motivated by this, several tensor decomposition based algorithms have been proposed. For example, Taguchi^16^ employed tensor decomposition to identify drug target genes using time-course gene expression profiles of human cell lines. Li and Ngom^17^ proposed a higher-order non-negative matrix factorization (HONMF) tool for classifying good or poor responders from a latent subspace corresponding to patients learned from HONMF. One limitation of this work is that the latent subspace may not have discriminative ability in classifying patients, since it is learned without accounting for the class-label information. Moreover, this method simply discards samples with missing values, causing unnecessary information loss.

Recently, Fukushima et al.^8^ developed an algorithm for joint gene selection and drug response prediction for time-course data. The method uses Elastic-Net (EN) to select a set of genes that show discrimination of patients’ drug responses throughout the treatment. The selected genes are then passed to a logistic regression (LR) classifier for drug response prediction. But in real applications, due to the existence of noise and missing values in the data, finding discriminative genes for all patients may be difficult. In fact, several studies have shown that it is more viable to find genes that have consistent discrimination in a subset of samples along the time series^18–20^. Therefore, relying only on discriminative gene selection but without modifying classification algorithms may not achieve satisfactory performance.

In this paper, we take a different approach for time-course drug response prediction. We hypothesize that a patient’s drug response at a given time-point can be inferred from the response at a previous time point. This means that not only the GEXs but also the past response results can be integrated to identify the drug response for a subsequent time point. We develop a REcursive Prediction (REP) algorithm to predict the drug response of samples using their time-course gene expression data and their drug response at previous time-points. REP has a built-in recursive structure that exploits the intrinsic time-course nature of the data through integrating past drug responses for subsequent prediction. In other words, in REP, not only the GEXs but also the past drug responses are treated as features for drug response prediction. Furthermore, by taking into consideration the intrinsic tensor structure of the time-course gene expression data and leveraging identifiability of low-rank tensors, REP can alleviate the noise corruption in GEX measurements, complete missing GEXs and even predict GEXs for subsequent time points. These features enable REP to evaluate drug response at any stage of a given treatment from some GEXs measured in the beginning of a treatment. Experiments on real data are included to demonstrate the effectiveness of the REP algorithm.

## Methods

**Figure 1:**
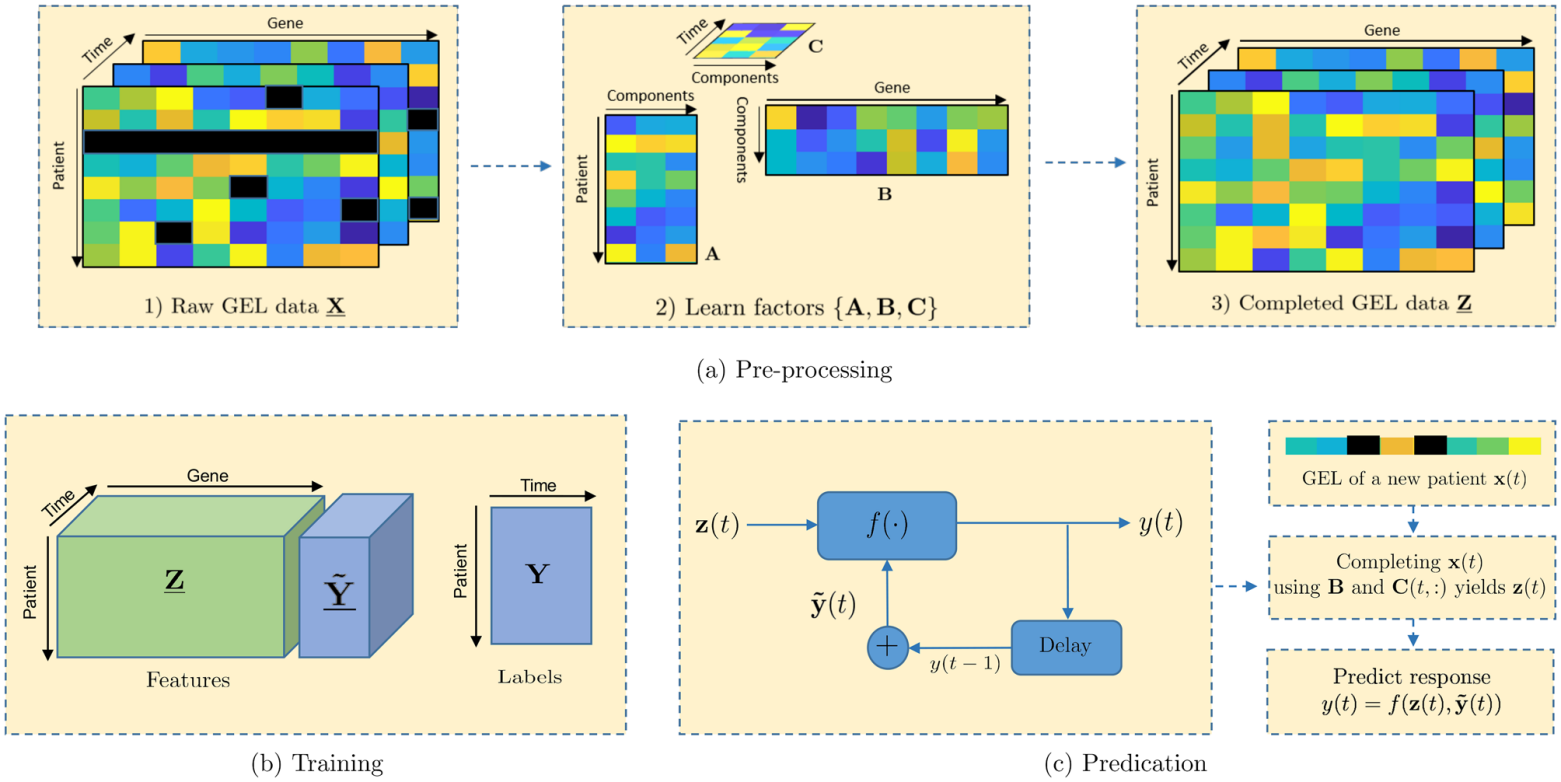
Sketch view of the proposed method. In (**a**), Step (1) shows the raw data ${\underline{\mathbf {X}}}$ with missing values marked as ‘black’; Step (2) shows the low-rank tensor factorization; Step (3) is the missing completion, where ${\underline{\mathbf {Z}}}= \mathscr {P}_{\Omega }({\underline{\mathbf {X}}}) + \mathscr {P}_{\Omega ^c}({\underline{\mathbf {G}}})$. In (**b**), it shows the composition of training data: features and labels. In (**c**), it shows the prediction for new patient at a specific time *t*.

Figure 1 sketches the idea behind the proposed REP algorithm, where the Fig. 1a–c show the pre-processing, model training and prediction of our method, respectively. The tensor structure of time-course gene expression data is shown in Fig. 1a. In the following, we explain them in more detail.

### Pre-processing

One major issue in using gene expression data for drug response prediction is the existence of missing values. To overcome this problem, we first impute the missing values during pre-processing. Various methods have been previously suggested for handling missing values, such as median-imputation^21^ and nearest neighbor imputation^22,23^. Instead, we employ a low-rank tensor model to fit the time-course gene expression dataset such that the missing values can be completed. Our supporting hypothesis is that genes never function in an isolated way, but oftentimes groups of genes interact together to maintain the complex biological process, which results in correlation in the GEX data^24^. We note that our low-rank tensor model suggests three factors that uniquely determine the values of GEXs, i.e., the factors corresponding to patient, gene and time, respectively (see Fig. 1). As we will see later, our model allows us to estimate the variation of GEX over time from a set of initial GEX measurements; these estimated values are then used to predict the time-course of drug response.

Towards this goal, we first assume (For high-enough but finite *F*, any patient-gene-time dataset can be expressed this way. See^25^ for a tutorial overview of tensor rank decomposition.) that each GEX is represented as a summation of *F* triple products from the latent factors of patient, gene and time, respectively. Let us denote $g_{ijk}$ as the *j*th GEX of patient *i* recorded at time *k*. Based on our assumption, we have

1 $$\begin{aligned} g_{ijk} =\sum _{f=1}^{F} a_{if}b_{jf}c_{kf} \end{aligned}$$

where $a_{if}$, $b_{jf}$ and $c_{kf}$ are the latent factors of patient, gene and time, respectively. Suppose that there are *J* genes measured over *K* time points. By varying the indices *j* and *k* in (1), the expression of the genes in all the time-points in patient *i* can be represented as

2 $$\begin{aligned} \mathbf {G}_{i} = \mathbf {B}\mathscr {D}_i(\mathbf {A})\mathbf {C}^T \in \mathbb {R}^{J\times K} \end{aligned}$$

where $\mathbf {A}\in \mathbb {R}^{I\times F}$, $\mathbf {B}\in \mathbb {R}^{J\times F}$, $\mathbf {C}\in \mathbb {R}^{K\times F}$. In this equation, $\mathscr {D}_i(\mathbf {A})$ represents a diagonal matrix holding the *i*th row of $\mathbf {A}$ as the main diagonal, which is a latent representation of the *i*th patient. We use $a_{if}$ to represent the (*i*; *f*)-entry of $\mathbf {A}$, $b_{jf}$ to represent the (*j*; *f*)-entry of $\mathbf {B}$ and $c_{kf}$ to represent the (*k*; *f*)-entry of $\mathbf {C}$.

Assume that there are *I* patients in the training set. After collecting $\{\mathbf {G}_1,\ldots ,\mathbf {G}_I\}$, we stack them in parallel along the patient-axis, which results in a GEX tensor that takes the form of

3 $$\begin{aligned} {\underline{\mathbf {G}}}:= \llbracket \mathbf {A},\mathbf {B},\mathbf {C}\rrbracket = \sum _{f=1}^F\mathbf {a}_f\circ \mathbf {b}_f\circ \mathbf {c}_f \in \mathbb {R}^{I\times J\times K} \end{aligned}$$

where $\circ$ is the outer product and $\mathbf {a}_f$ is the *f*th column of $\mathbf {A}$, and likewise for $\mathbf {b}_f$ and $\mathbf {c}_f$. Here, we assume that ${\underline{\mathbf {G}}}$ is the noiseless GEX data and ${\underline{\mathbf {X}}}$ is the corresponding noisy data with missing values. The relationship between ${\underline{\mathbf {G}}}$ and ${\underline{\mathbf {X}}}$ is described as

4 $$\begin{aligned} \mathscr {P}_\Omega ({\underline{\mathbf {X}}}) = \mathscr {P}_\Omega ({\underline{\mathbf {G}}}) + \mathscr {P}_\Omega ({\underline{\mathbf {N}}}) \end{aligned}$$

where ${\underline{\mathbf {N}}}$ is the noise in the data, $\Omega$ is the index set of the observed GEXs in ${\underline{\mathbf {X}}}$, and $\mathscr {P}_{\Omega }$ is the operator that keeps the entries in $\Omega$ and zeros out the others.

The model in (2) indicates that the gene and time factors (i.e., $\mathbf {B}$ and $\mathbf {C}$) are identical for different patients, and the variability among patients is captured by $\mathbf {A}$. In other words, given $\mathbf {B}$ and $\mathbf {C}$, each row of the patient factor matrix $\mathbf {A}$ uniquely determines the GEXs of the corresponding patient. As we will see later, our model is able to predict unseen GEXs, which also enables to prescreen the drug response for different stages of a treatment.

Assuming non-negative GEXs, (Due to some preprocessing steps such as z-score normalization, the GEX values can be negative. To facilitate our method, we undo these preprocessing steps or use the raw dataset.) we can use non-negative tensor factorization to compute missing GEX values:

5 $$\begin{aligned} \min _{\mathbf {A},\mathbf {B},\mathbf {C},{\underline{\mathbf {G}}}}&\left\| {\underline{\mathbf {G}}}- \llbracket \mathbf {A},\mathbf {B},\mathbf {C}\rrbracket \right\| _F^2 + \mu \left( \Vert \mathbf {A}\Vert _F^2 + \Vert \mathbf {B}\Vert _F^2 + \Vert \mathbf {C}\Vert _F^2\right) \nonumber \\ \mathrm {s.~t.}\;&\;\mathscr {P}_{\Omega }({\underline{\mathbf {G}}}) = \mathscr {P}_{\Omega }({\underline{\mathbf {X}}}), \mathbf {A}\ge 0, \mathbf {B}\ge 0, \mathbf {C}\ge 0 \end{aligned}$$

where many sophisticated algorithms are applicable to optimize (5), e.g., block coordinate descent^25,26^. Intuitively, (5) seeks to identify the lowest rank solution $\mathbf {G}$ that best matches the observations ${\underline{\mathbf {X}}}$. The regularization terms are added to further encourage low rank and prevent over-fitting. When (5) is solved, we complete the GEX data through

6 $$\begin{aligned} {\underline{\mathbf {Z}}}= \mathscr {P}_{\Omega }({\underline{\mathbf {X}}}) + \mathscr {P}_{\Omega ^c}({\underline{\mathbf {G}}}) \end{aligned}$$

where $\Omega ^c$ contains the indices of missing values in ${\underline{\mathbf {X}}}$.

### Training

The effects of drugs are usually cumulative over time^27^, i.e., drug doses taken in the past will affect the current response. This implies that the drug response in the past time-points may help predict the current response. Based on this hypothesis, we propose a recursive prediction algorithm, henceforth referred to as REP for simplicity, which enables to integrate past drug response records with gene expression values for subsequent drug response predictions. Figure 1c shows an overview of REP’s pipeline, where drug responses $\{y(0),\ldots ,y(t-1)\}$ in the previous time stages are integrated with the gene expression information $\mathbf {z}_{t}$ for predicting the current response *y*(*t*). Here, we accumulate the historical responses by concatenating them into a new vector as

7 $$\begin{aligned} {\tilde{\mathbf {y}}}(t) = [y(t-1), y(t-2), \ldots , y(0), 0, \ldots , 0]^T\in \mathbb {R}^{K-1} \end{aligned}$$

which is then fed back as an input feature for subsequent drug response prediction. Therefore, at time *t*, the output of the predictor depends not only on the GEX at that time point, but also the previously observed drug responses. We always insert the drug response from the most recent time point into the first element of ${\tilde{\mathbf {y}}}(t)$, so that the model can capture a sense of time, and learn from recent/emerging trends in drug response.

For the *i*th patient at time *t*, we concatenate ${\tilde{\mathbf {y}}}_{i,t}$ and the corresponding GEX vector $\mathbf {z}_{i,t}$ together, where ${\tilde{\mathbf {y}}}_{i,t}$ denotes the historical responses of patient *i* at time *t*. We then pass $\mathbf {z}_{i,t}$ and $\mathbf {y}_{i,t}$ to a predictor $f(\cdot )$ to predict drug response, i.e.,

8 $$\begin{aligned} y_{i,t} = f(\mathbf {z}_{i,t}, {\tilde{\mathbf {y}}}_{i,t}) \end{aligned}$$

where $f(\cdot )$ can be trained by minimizing the following cost function

9 $$\begin{aligned} L(\varvec{\theta }) = \frac{1}{IK}\sum _{i=1}^{I}\sum _{t=1}^K \ell (f(\mathbf {z}_{i,t}, {\tilde{\mathbf {y}}}_{i,t}), y_{i,t}) + \lambda r(\varvec{\theta }) \end{aligned}$$

where $\varvec{\theta }$ contains the parameters of the predictor, $\ell (\cdot )$ is the loss function of a classifier such as hinge loss and cross-entropy loss, $r(\cdot )$ is a regularizer that imposes a certain structure on $\varvec{\theta }$, and $\lambda \ge 0$ is a regularization parameter. In the literature, popular regularizers include $r(\varvec{\theta }) = \Vert \varvec{\theta }\Vert _2^2$, $r(\varvec{\theta }) = \Vert \varvec{\theta }\Vert _0$, $r(\varvec{\theta }) = \Vert \varvec{\theta }\Vert _1$ and $r(\varvec{\theta })=1_+(\varvec{\theta })$, i.e., the indicator function of the non-negative orthant.

Our main idea is to feed back the historical drug responses and then combine them with GEX values to predict the drug response in the future. This is the major difference between our method and the state-of-the-art algorithms: prior art ignored the previous drug response outputs. Therefore, the training set for our method is created in a slightly different way. Recall that at each time point, we stack the historic drug responses into a vector. For any patient in the training set, we can further concatenate all such prior response vectors for *K* time points together, which yields a feedback matrix for patient *i* as

10 $$\begin{aligned} {\tilde{\mathbf {Y}}}_i = \begin{bmatrix} {\tilde{\mathbf {y}}}_{1,1}&{\tilde{\mathbf {y}}}_{1,2}&\cdots&{\tilde{\mathbf {y}}}_{1,K} \end{bmatrix}^T \in \mathbb {R}^{K\times (K-1)}. \end{aligned}$$

Furthermore, we can create a tensor $\underline{{\tilde{\mathbf {Y}}}}\in \mathbb {R}^{I\times K\times (K-1)}$ by concatenating all $\{{\tilde{\mathbf {Y}}}_1, \ldots , {\tilde{\mathbf {Y}}}_I\}$ together, where $\underline{{\tilde{\mathbf {Y}}}}(i, :, :) = {\tilde{\mathbf {Y}}}_i$. Finally, the features in the training set are formed by concatenating ${\underline{\mathbf {Z}}}$ and $\underline{{\tilde{\mathbf {Y}}}}$ along the gene-axis as shown in the left-bottom corner of Fig. 1b, and the training labels are

11 $$\begin{aligned} \mathbf {Y}= \begin{bmatrix} y_{1,1} &{} y_{1,2} &{} \cdots &{} y_{1,K} \\ y_{2,1} &{} y_{2,2} &{} \cdots &{} y_{2,K} \\ \vdots &{} &{} &{} \\ y_{I,1} &{} y_{I,2} &{} \cdots &{} y_{I,K} \end{bmatrix}. \end{aligned}$$

It is also important to mention that our method can predict either binary or non-binary drug responses, e.g., continuous values. When the drug response is binary, the predictor $f(\mathbf {z}_{i,t},{\tilde{\mathbf {y}}}_{i,t})$ will typically be a classifier. When the drug response is continuous, $f(\mathbf {z}_{i,t},{\tilde{\mathbf {y}}}_{i,t})$ will be a regression algorithm.

It is important to note that our approach is really a framework that is applicable no matter what is the choice of the final classification or regression algorithm. Nevertheless, for the purposes of exemplifying and illustrating the merits of our proposed framework, we are particularly interested in support vector machines (SVM and SVR, for classification and regression, respectively), which have shown promising performance in this type of task (cite a few past papers using SVM for this task here). We set $\varvec{\theta }=[\mathbf {u}^T,\mathbf {v}^T]^T$ and $\ell (\cdot )$ to be the hinge loss, resulting in

12 $$\begin{aligned} \min _{\mathbf {u},v,b}&\frac{1}{IK}\sum _{i=1}^{I}\sum _{t=1}^K\max \left( 0, 1 - y_{i,t}(\mathbf {u}^T\mathbf {z}_{i,t} + \rho \mathbf {v}^T {\tilde{\mathbf {y}}}_{i,t} + b)\right) + \frac{\lambda }{2}\left( \Vert \mathbf {u}\Vert _2^2+\Vert \mathbf {v}\Vert ^2\right) \nonumber \\ \mathrm {s.~t.}&[\mathbf {u}^T,\mathbf {v}^T]^T\in \mathscr {C} \end{aligned}$$

where *b* is the intercept, $\mathscr {C}$ denotes a convex set such as $\ell _1$-ball for feature selection and $\rho$ represents the importance of the response at a previous time point on the subsequent one.

In our formulation, the drug response feedback plays an important role and it can be viewed as a “must-have” feature. In SVMs, we penalize the two-norm of the linear weights equally—the implicit assumption being that features have similar powers. In our context, however, the GEX values are much larger than the drug response labels which are either 1 or $-1$. As a result, the GEX values are likely to end up playing a more significant role in the prediction—simply because we cannot scale the labels up to any meaningful level, due to the regularization term. To compensate for this imbalance, in the above formulation, we introduce fixed weight $\rho$ in the cost function. In practice, we recommend to choose a relatively large $\rho$.

### Drug response prediction

Our method can predict the drug response values for a new patient at any time point. Specifically, given the GEXs of a new patient at time *t*, i.e., $\mathbf {x}(t)$, we first check if there are missing values. If so, we employ the factors $\mathbf {B}$ and $\mathbf {C}$ to complete $\mathbf {x}(t)$. Let us denote ${{\bar{\Omega }}}$ and ${{\bar{\Omega }}}^c$ as the sets of indices of the observed and missing elements in $\mathbf {x}(t)$. According to our model in (1), $\mathbf {x}(t)$ can be uniquely determined by $\mathbf {B}$, $\mathbf {C}$ and an unknown vector $\mathbf {a}$—a latent representation of this new patient. Thus, for the expression level of the *j*th gene at time *t*, we have

13 $$\begin{aligned} x_j(t)&= \begin{bmatrix} b_{j1}&\cdots&b_{jF} \end{bmatrix} \begin{bmatrix} a_{1} &{} &{} \\ &{}\ddots &{} \\ &{} &{}a_{F} \end{bmatrix} \begin{bmatrix} c_{t1} \\ \vdots \\ c_{tF} \end{bmatrix} + \mathbf {n}\nonumber \\&= \left( \mathbf {C}(t,:)\odot \mathbf {B}(j,:)\right) \mathbf {a}+ n_j,~\forall j\in {{\bar{\Omega }}} \end{aligned}$$

where $n_j$ is the additive noise which is assumed as Gaussian distributed, $\odot$ is the Khatri-Rao (column-wise Kronecker) product, and $\mathbf {B}(j,:)$ and $\mathbf {C}(t,:)$ denote the *t*th row of $\mathbf {B}$ and $\mathbf {C}$, respectively.

Since $\mathbf {B}$ and $\mathbf {C}$ are known, the problem of estimating $\mathbf {a}$ can be formulated as

14 $$\begin{aligned} {{\hat{\mathbf {a}}}} = \arg \min _{\mathbf {a}\ge 0} \sum _{j\in {{\bar{\Omega }}}} \left( x_j(t) - \left( \mathbf {C}(t,:)\odot \mathbf {B}(j,:)\right) \mathbf {a}\right) ^2 \end{aligned}$$

which is a non-negative least squares (NLS) problem and can be optimally solved. We note that to obtain a unique estimate ${{\hat{\mathbf {a}}}}$, the number of available gene expression entries in $\mathbf {x}(t)$ should be $\ge F$. The GEX vector of the patient is then estimated as

15 $$\begin{aligned} \mathbf {g}(t) = \left( \mathbf {C}(t,:)\odot \mathbf {B}\right) {{\hat{\mathbf {a}}}} \end{aligned}$$

which leads to a completed GEX vector

16 $$\begin{aligned} \mathbf {z}(t) = \mathscr {P}_{{{\bar{\Omega }}}}(\mathbf {x}(t)) + \mathscr {P}_{{{\bar{\Omega }}}^c}(\mathbf {g}(t)). \end{aligned}$$

The vector $\mathbf {z}(t)$ together with the cumulated historical drug response ${\tilde{\mathbf {y}}}(t)$, are the input data for our predictor $f(\cdot )$. We estimate the drug response of this patient at time *t* via

17 $$\begin{aligned} {\hat{y}}(t) = f\left( \mathbf {z}(t), {\tilde{\mathbf {y}}}(t) \right) . \end{aligned}$$

It is crucial to mention that in some cases, there might be missing labels in the testing set, such that ${\tilde{\mathbf {y}}}(t)$ cannot be constructed. To handle this scenario, we can use the predicted labels instead of the missing ones to construct ${\tilde{\mathbf {y}}}(t)$. More specifically, we start from $t=0$ and predict *y*(0), which is used to construct ${\tilde{\mathbf {y}}}(1)$. Then we use ${\tilde{\mathbf {y}}}(1)$ to predict the response at $t=1$, so on and so forth.

#### Predicting unseen GEXs

Previously, we have explained how to predict drug response for patients at a certain time point. However, in practice, we are more interested in knowing the drug response of a few time-points in the future from the beginning of a treatment. This requires to know the GEXs of all time points up to the one of interest a priori, which is impossible in practice. In this subsection, we provide an efficient solution that allows to predict the unseen GEXs.

Recall that in our model, the GEX of a patient is determined by three factors, i.e., the latent representation of patient—$\mathbf {a}$, the time evolution factor—$\mathbf {B}$ and the gene factor matrix—$\mathbf {C}$, where $\mathbf {a}$ is different for patients, and needs to be estimated for the new patient. On the other hand, $\mathbf {B}$ and $\mathbf {C}$ are common gene and time evolution bases that reflect different types of patients, as determined from historical patient data—the training data. Therefore, the problem boils down to the estimation of $\mathbf {a}$ from the initial GEXs of the new patient. We can simply substitute $t=1$ in (14) to find ${{\hat{\mathbf {a}}}}$. Finally, the GEXs for the remaining time points are estimated as

18 $$\begin{aligned} {\hat{\mathbf {x}}}(t) = \left( \mathbf {C}(t,:)\odot \mathbf {B}\right) {{\hat{\mathbf {a}}}}, \quad \forall t= 2,\ldots ,K. \end{aligned}$$

Now we have estimated the unseen GEXs for $t\ge 2$, which allows us to predict drug response values for the whole duration of the treatment. We start from ${{\hat{\mathbf {x}}}}(1)$ and estimate the drug response for $t=1$ as

19 $$\begin{aligned} {\hat{y}}(1) = f({\hat{\mathbf {x}}}(1), {\tilde{\mathbf {y}}}(1)) \end{aligned}$$

where ${\tilde{\mathbf {y}}}(1) = 0$. When ${\hat{y}}(1)$ is available, we set ${\tilde{\mathbf {y}}}(2) = {\hat{y}}(1)$. With the GEX estimate ${\hat{\mathbf {x}}}(2)$ from (18), we can predict ${\hat{y}}(2) = f({\hat{\mathbf {x}}}(2), {\tilde{\mathbf {y}}}(2))$, and so forth for the other time points.

##### Remark 1

Note that here we substitute predicted drug responses for the unseen drug responses. Clearly, when actual drug responses for past time ticks are available, they should be used. We only do the substitution here for a preliminary assessment of how well a patient is likely to respond over time, before the beginning of treatment—which is naturally a more ambitious goal.

## Experiments

In this section, we provide some numerical experiments to showcase the effectiveness of REP for drug response prediction from time-course gene expression data. We examine two tasks: classification on binary drug response and regression on continuous drug response.

### Dataset

We consider two datasets to evaluate the performance of our method.

- The first dataset used is the interferon (IFN)-$\beta$ time-course dataset which is available in the supplementary of^15^. The dataset was collected from 53 Multiple Sclerosis (MS) patients who received IFN-$\beta$ treatment for 2 years. The gene expression data (microarray) was obtained from peripheral blood mononuclear cells of the patients, which contained the expression levels of 76 pre-selected genes over seven stages (i.e., time-points) of the treatment, where the time lag between two adjacent time points was 3 months in the first year, and 6 months in the second year. The responses to the therapy were measured at each time point using the expanded disability status scale (EDSS) which is a method of quantifying disability in multiple sclerosis and monitoring changes in the level of disability over time^28^. Note that EDSS values in the dataset are between 0 and 7, where the higher EDSS values reflect more severe disability. Except for the EDSS at the initial time point, the others were measured after the IFN-$\beta$ injection at each time point. Therefore, we focus on the prediction of EDSS after $t=1$. In addition to EDSS, whether a patient had good or poor response to each treatment was also recorded, for each patient—and this is the indicator that we seek to predict in our classification experiments. The percentage of good patient responses to individual treatments was 58.5%; the remaining 41.5% responses were poor, on average, across the patient population considered. There are also missing values in this dataset, where most missing values were caused by the absence of patients at some stages. Only 27 patients had records for all stages, while the other 26 patients missed at least one stage, which resulted in the entire GEXs as well as the drug response at that stage being missed. In the following experiments, unless specified otherwise, we employ the 27 full records to evaluate the performance of algorithms, where the final GEX data is of size $27\times 7\times 76$ and the response data is of size $27\times 7$.
- The second dataset is from a Gene Expression Omnibus (GEO) record GSE24427^29^ also corresponding to MS. In the dataset, there are 16 female patients and 9 male patients who received IFN-$\beta$ therapy for 24 months. During the treatment, the RNA expression values were measured five times: at baseline (before first IFN-$\beta$ injection), 2 days (before second IFN-$\beta$ injection), and 1 month (before month-1 IFN-$\beta$ injection), 12 months (before month-12 IFN-$\beta$ injection), and 24 months (before month-24 IFN-$\beta$ injection), respectively. The EDSS values were measured four times: at baseline, after 1 year, 2 years and 5 years of the initial injection, respectively. We use (1) the RNA expressions measured before month-12 to predict the EDSS measured after 1 year treatment, (2) the gene expressions measured before month-24 to predict the EDSS measured after 2 years treatment. There are 47,522 gene probes in this dataset. We employed the python package mygene (mygene: https://mygene.info/) to map the probes to gene names, which yielded 19,565 gene names.

Unlike the first dataset, we do not have binary drug response in the second dataset. Therefore, we focus on the prediction of binary drug response on the first dataset (whether or not a patient will have good or poor response), while the prediction of EDSS for both datasets is viewed as a regression task, because EDSS is an ordinal variable (predicting a 6 as a 7 is better than predicting a 6 as a 3; thus mean absolute error and room mean squared error make sense as performance metrics).

### Methods for comparison

We examine the predictive ability on the prediction of binary drug response and ordinal EDSS response. For the binary case, we apply a number of classifiers including two linear models (EN-LR^8^ and SVM), one nonlinear model (*K*-nearest neighbors (KNN)^30^), and a probabilistic graphical model (discriminative loop hidden Markov model (dl-HMM)^14^) to real-world time-course data. We did not include SVM with nonlinear kernels (e.g. Gaussian), since its performance was inferior compared to the linear kernel. Note that EN-LR and dl-HMM were specifically designed for prediction of drug response values based on time-course gene expression data, while SVM and KNN are widely used classification methods.

For ordinal prediction, we implement Elastic Net, Support Vector Regression (SVR) with radial basis function (rbf) krenel, Random Forest and KNN on the two datasets. All methods are implemented via the Python sklearn package with version 0.0. We use default settings for Elastic Net and SVR algorithm. For Random Forest, we set the number of trees in the forest to 20. For KNN, we set the number of neighbors to 10. For each dataset, we first create two versions of training and testing sets, where one is with the drug response feedback described in Fig. 1 and the other one is without feedback. We use REP-ElasticNet, REP-SVR, REP-RandomForest and REP-KNN to denote the respective algorithms with drug response feedback.

### Evaluation metric

For classification, we use prediction accuracy (ACC) and area under receiver operating characteristic (ROC) curve (AUC) to evaluate the performance of REP, where ACC is defined as:

$$\begin{aligned} \mathrm {ACC} = \frac{\mathrm{TP + TN}}{\mathrm{TP + FP + FN + TN}}. \end{aligned}$$

In the equations above, TP, FP, FN and TN stand for the number of true positives, false positives, false negatives and true negatives, respectively. The calculation of AUC is based on the ROC, which plots TP versus FP. Here, each pair of TP and FP are obtained by comparing the score of a classifier with a varying threshold.

For regression, we use mean squared error (MSE) and mean absolute error (MAE) to evaluate the performance, where MSE and MAE are defined as

$$\begin{aligned} \mathrm {MSE}&= \frac{1}{MN}\sum _{m=1}^M\sum _{n=1}^{N}\Vert \mathbf {y}_m - {\hat{\mathbf {y}}}_m\Vert _2^2 \\ \mathrm {MAE}&= \frac{1}{MNT}\sum _{m=1}^M\sum _{n=1}^{N}\sum _{t=1}^T\left| y_m(t) - {\hat{y}}_m(t)\right| \end{aligned}$$

where *M* is the number of samples in the testing set, *N* is the number of Monte-Carlo tests, $\mathbf {y}_m$ denotes the ground-truth drug response of the *m*th testing sample and ${\hat{\mathbf {y}}}_m$ is its estimate with $y_m(t)$ and ${\hat{y}}_m(t)$ being their values at time *t*, respectively.

We report performance for all methods by using the same training, validation and testing sets. Specifically, we employ leave-one-out cross validation (LOO) for testing, where at each fold, we split 27 patients into a training set with 26 patients and a testing set with one patient. We then hold the testing set and randomly split the training set into two parts, where the first part has 25 patients and the second part has one patient, i.e., the validation set. We train models on the first part and tune hyper-parameters on the second part. Note that for each algorithm, we select its best hyper-parameters—those that yield the highest prediction accuracy on the validation set. Finally, we apply the selected model to the testing set. For a fair comparison, in all experiments, we apply the same missing value imputation method to all algorithms.

For REP-SVM, its hyper-parameters include $\lambda$ and $\rho$ in (12), which are selected from $\lambda =\{0.1, 0.5\}$ and $\rho \in \{50,100\}$. For the standard SVM method, it solves the following problem:

20 $$\begin{aligned} \min _{\mathbf {u},b}&~ \frac{1}{IK}\sum _{i=1}^{I}\sum _{t=1}^K\max \left( 0, 1 - y_{i,t}(\mathbf {u}^T\mathbf {z}_{i,t} + b)\right) + \frac{\lambda }{2}\Vert \mathbf {u}\Vert _2^2 \end{aligned}$$

where $\lambda$ is tuned from $\{0.01,0.1,1,10\}$; for EN-LR, we set $\alpha =0.5$ which is a hyper-parameter balancing the ridge and LASSO regularizations; for KNN, the number of neighbors is selected from $\{3,5,8,10\}$. After that, we apply the trained classifier to the testing data to calculate ACC. We implemented REP-SVM, EN-LR, SVM and KNN in Python 3.7. Since the authors of dl-HMM have published their MATLAB codes (http://www.cs.cmu.edu/~thlin/tram/), we used their MATLAB implementation for our comparison. The hyper-parameter for dl-HMM is the number of hidden states, which is chosen from $\{2,3,4\}$.

### Results

#### Parameter selection for tensor completion

**Figure 2:**
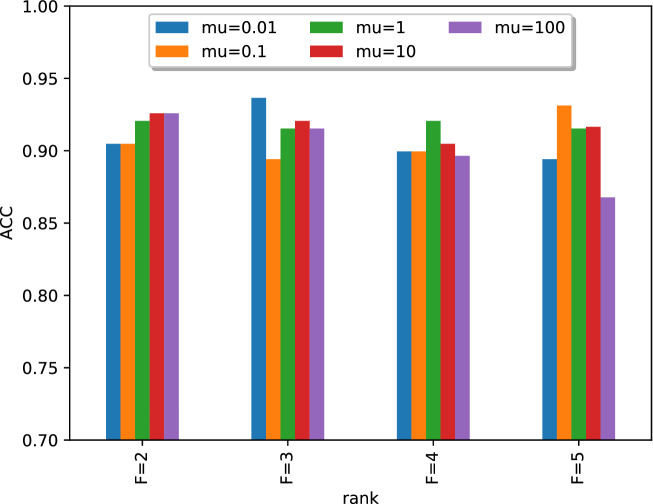
Choosing *F* and $\mu$ for the tensor completion algorithm.

We first study how the hyper-parameters *F* and $\mu$ in (5) affect the prediction performance. The percentage of missing values in GEXs is fixed at 5%. We vary *F* from 2 to 5 and $\mu$ from 0.01 to 100, and report ACC of REP-SVM on the classification task, i.e., predicting good or poor responder. The ACC is calculated using leave-one-out (LOO) cross-validation, where in each fold, we select one patient’s record as a testing set that contains a $7\times 76$ GEX matrix and a response vector with length 7, while the remaining 26 records are assigned to the training set. It can be seen in Fig. 2 that $F\le 3$ produces better results than $F\ge 4$ in general, especially when $\mu \ge 1$.

#### Performance evaluation on binary drug response

We now compare the performance of the five algorithms in terms of prediction accuracy and AUC. In the raw data, the missing values in ${\underline{\mathbf {X}}}$ are only 0.23%. For all the methods, the missing GEXs were completed using non-negative tensor completion in Section II-A, where we set $F=3$ and $\mu =1$. It can be seen in Table 1 (i.e., the rows with % miss as 0.23) that REP-SVM achieves a higher prediction accuracy compared to other methods and its performance is followed by the SVM and EN-LR algorithms. The KNN and dl-HMM algorithms have relatively low accuracy. We note that REP-SVM takes a similar formulation as the SVM. However, REP-SVM is 2.5% more accurate than SVM, which implies that the recursive structure in REP-SVM is helpful in improving the prediction accuracy.

**Table 1:** ACC & AUC versus percentage of missing values, where all metrics are obtained via LOO CV on the 27 patients with seven time points in Dataset1.

Metrics	Miss $\%$	REP-SVM	dl-HMM	EN-LR	SVM	KNN
ACC	0.23	0.925	0.851	0.882	0.898	0.719
	5	0.912	0.818	0.865	0.857	0.701
	10	0.886	0.809	0.878	0.855	0.685
	15	0.876	0.818	0.844	0.837	0.721
	20	0.872	0.781	0.830	0.825	0.718
AUC	0.23	0.971	0.875	0.927	0.968	0.413
	5	0.963	0.769	0.923	0.940	0.472
	10	0.951	0.744	0.926	0.934	0.461
	15	0.942	0.723	0.875	0.907	0.454
	20	0.941	0.710	0.878	0.886	0.497

We sought to determine the effect of missing values on the performance of these methods. For this purpose, we randomly sampled the GEX data and hid the selected entries. As the percentage of missing values increases, all methods suffer performance loss, but REP-SVM’s ACC and AUC remain the highest in all cases (see Table 1). We highlight that when the percentage of missing values is 20%, REP-SVM has ACC close to 0.872 and AUC greater than 0.941. EN-LR outperforms the classical SVM method in many cases. When the percentage of missing values increases, the performance of EN-LR and SVM drop significantly, while that of REP-SVM still remains at a high level. For example, when the percentage of missing increases to 15%, the ACC of EN-LR drops to 0.844 and that of SVM drops to 0.837, but ours is 0.887, which indicates that REP-SVM is more robust against missing values. We mention that in this experiment, the ratio of the positive and negative classes is 19/8. So the percentage of the positive class is about 70.4%. We found that most of the predicted labels of KNN were positive, meaning that it cannot distinguish the negative class. The scores produced by KNN were not good enough to separate the two classes. This is why KNN yields seemingly reasonable accuracy but low AUC.

In this example, we evaluate the performance comparison on all patients in Dataset1. As we have mentioned before, there are 26 patients that do not have seven time points records, so they cannot be used in the training step of REP-SVM. Therefore, we use them for testing, and the training is based on the 27 patients with full temporal records, where we set $F=3$ and $\mu =1$. Note that in the testing data, approximately 18.1% of the GEX values and 22% drug response labels are missing. Here, the missing GEXs are completed through our non-negative tensor completion. We calculate the ACC and AUC based on the known drug response labels. The results are shown in Table 2. We see that REP-SVM outperforms the other competitors in both accuracy and AUC, where it achieves about 0.790 in ACC and 0.884 in AUC. EN-LR has the second best performance and is followed by SVM in terms of accuracy, but EN-LR has higher AUC than SVM. In this case, the distributions of missing values in the training and testing sets are very different, where the percentage of missing values in training set is about 0.23% but that in the testing set is about 18.1%. Recall that in Table 1, where the missing values were randomly assigned through a uniform distribution, REP-SVM, EN-LR and SVM have higher ACC and AUC than the results in Table 2, even though the percentage of missing values reaches 20%. This indicates that the distribution of missing values in training and testing sets may affect the performance of drug response predictors.

**Table 2:** ACC and AUC comparison using all patients’ data in Dataset1, where the training set contains 27 patients with seven time points and testing set consists of the remaining 26 patients with less than seven time points.

Metrics	REP-SVM	dl-HMM	EN-LR	SVM	KNN
ACC	0.790	0.651	0.751	0.775	0.549
AUC	0.884	0.687	0.805	0.853	0.547

Percentage of missing values in the training set is 0.23% and that in the testing set is 18.1%.

We have shown that under the same completion algorithm, REP-SVM has better performance than the methods, but one may wonder if the same conclusion holds for other types of completion. To answer this question, we further compare REP-SVM, EN-LR, and SVM with mean, median, and KNN imputation. The results are shown in Table 3. We see that all predictors with tensor completion achieve the highest ACC and AUC compared to the standard imputation methods. We also note that REP-SVM continues to have the best performance even when used with less sophisticated imputation methods.

**Table 3:** ACC & AUC versus percentage of different imputation methods using Dataset1, where the training set contains 27 patients with seven time points and testing set consists of the remaining 26 patients with less than seven time points.

Metrics	Imputation method	REP-SVM	EN-LR	SVM
ACC	Tensor completion	0.790	0.751	0.775
	Median	0.724	0.713	0.721
	Mean	0.736	0.706	0.722
	KNN	0.738	0.723	0.721
AUC	Tensor completion	0.884	0.805	0.853
	Median	0.838	0.743	0.812
	Mean	0.835	0.739	0.812
	KNN	0.837	0.748	0.798

Percentage of missing values in the training set 0.23% and that in the testing set is 18.1%.

**Figure 3:**
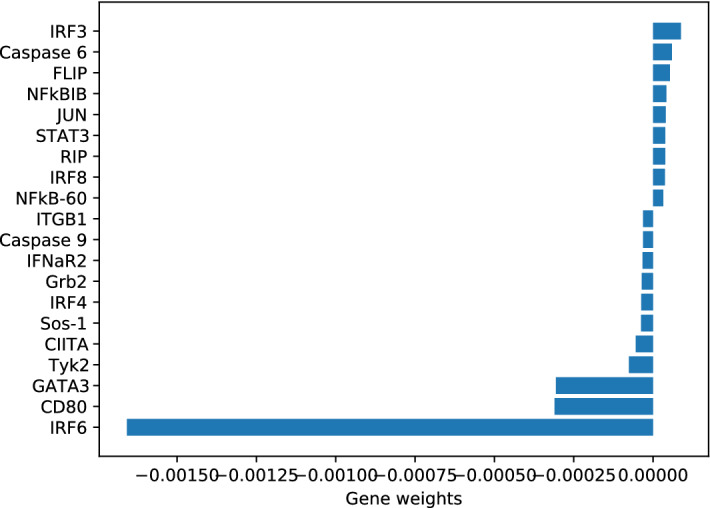
Top 20 genes in Dataset1 selected by REP-SVM according to their weights in $\mathbf {u}$ in (12) learned from the training set corresponding to Dataset1.

Figure 3 shows the top 20 genes selected by REP-SVM. Note that we run REP-SVM ten times on the 27 patients with full temporal records, average the weights corresponding to the genes and then rank the weights to generate the gene ranking. The genes IRF3, IRF4, IRF6 and IRF8 belong to the interferon regulatory transcription factor (IRF) family, which is critical for induction of type I (IFN-$\alpha /\beta$) and type III (IFN-$\beta$) IFN expression^31–33^.

#### Performance evaluation on ordinal drug response

Next, we evaluate the performance of the proposed method on the prediction of EDSS. Since the number of genes is much larger than the number of patients, it is challenging to perform regression directly on such an underdetermined dataset. To handle this issue, we select the genes which are also in the first dataset. This results in 42 overlapped genes (see Supplementary Table S1). Therefore, in the following experiments, we only used the selected 42 genes. Note that all methods use the same genes for regression, so reducing the number of genes will not affect the fairness. The results are shown in Table 4, where the percentage of missing values of GEX in Dataset1 is 0.23% and that in Dataset2 is 0. In both datasets, all methods with drug response feedback outperform their respective version without feedback in terms of MAE and MSE.

**Table 4:** Regression performance comparison using Dataset1 and Dataset2.

	Method	MAE	MSE	Method	MAE	MSE
Dataset1	REP-ElasticNet	0.919	1.504	ElasticNet	1.208	2.683
	REP-KNN	0.839	1.305	KNN	1.345	3.236
	REP-RandomForest	0.809	1.098	RandomForest	1.294	2.946
	REP-SVR	0.727	0.891	SVR	1.282	3.110
Dataset2	REP-ElasticNet	0.934	1.237	ElasticNet	1.007	1.447
	REP-KNN	0.658	0.780	KNN	1.129	1.798
	REP-RandomForest	0.930	1.265	RandomForest	1.095	1.753
	REP-SVR	0.660	0.792	SVR	1.146	1.897

Table 4 shows the overall performance comparison by averaging all time points. One may also be interested in the performance of REP at each time point. In Supplementary Tables S2–S5, we summarized the performance of the predictors at each time point, where we can see that REP-ElasticNet, REP-KNN, REP-RandomForest and REP-SVR achieved better performance than Elastic Net, KNN, Random Forest and SVR, respectively, in all cases. We would like to demonstrate that the REP framework can produce more accurate results for patients with not only constant drug responses but also time-varying drug responses. In Dataset1, there is only one patient has constant drug responses at different time points while the other 26 patients have time varying drug responses, i.e., there is at least one switch in the responses between two time points. In Dataset2, there are 14 patients with constant drug responses and 11 patients with time varying drug responses. For each dataset, we calculated the MSE and MAE based on two cases: one is based on patients with constant drug responses and the other one is based on patients with time-varying drug responses. The results are summarized in Supplementary Table S6, where REP-ElasticNet, REP-KNN, REP-RandomForest and REP-SVR performed better in all cases. In the constant response case in Dataset1, all methods had large MAE and MSE. The reason is that all training samples were with time-varying drug responses such that the algorithms were not trained well for the constant drug response case. In the second dataset, since we had more cases of constant drug responses in the training set, REP based algorithms worked much better. This indicates again that standard predictors with the REP framework can predict the drug responses more accurately [see Supplementary Figs. S1 and S2, where the actual and predicted drug responses (from the predictors) of different patients were plotted].

The above experiments are all based on the actual GEX values with only a small portion of them imputed. In the last experiment, we assume that in the testing set, only the GEX values at the initial time point have been observed. The training set and the way of imputing the missing values therein are kept the same as in the previous experiment in Table 4. Our task is to predict future drug responses for the remaining time points at an initial time point. In this case, the GEX values for the future time points are entirely missing. To handle this issue, we first implement the tensor completion method to learn the latent factor $\mathbf {B}$ for GEX and another latent factor $\mathbf {C}$ corresponding to the *T* time points *from the training set*. Then according to (1), given the GEX vector of the *i*th patient at the initial time point, we can estimate his/her latent representation denoted by $\mathbf {a}_i$, by solving a non-negative last squares problem, i.e., $\min _{\mathbf {a}_i\ge 0} \Vert \mathbf {g}_0 - \mathbf {B}\mathrm {diag}(\mathbf {c}_0)\mathbf {a}_i\Vert _2^2$, where $\mathbf {g}_0$ is the observed GEX values at $t=0$ and $\mathbf {c}_0$ is the first row of the estimated temporal factor matrix $\mathbf {C}$ corresponding to $t=0$. This problem is convex and it can be optimally solved. Then we can substitute $\mathbf {a}_i$ into (2) to predict the entire GEX matrix for the *i*th patient and we use the second to the last column of $\mathbf {G}$ for prediction, where the *t*th column in $\mathbf {G}_i$ represents the predicted GEX values at time *t*. Furthermore, we do not have any information about the drug responses for the future except for the one at $t=0$, and thus unable to feed back the actual drug response at $t \ge 1$ for subsequent prediction. We have mentioned previously that for such cases, we can feed back the predicted drug responses for future prediction if a previous drug response is unavailable. Therefore, in the testing step, we start from $t=1$ where we can use the GEX values and EDSS value at $t=0$ to predict $t=1$. Then we use this estimated drug response and the estimated GEX at $t=1$ to predict $t=2$, so on and so forth.

Table 5 shows the results, where the column ‘Using estimated GEX’ stands for using estimated GEXs and feeding back estimated drug responses for subsequent prediction, while ’Using actual GEX’ stands for using actual GEXs and feeding back actual drug responses. It can be seen in Table 5 that predictors using the predicted GEX with the feedback of predicted drug responses have slightly worse performance than using the actual GEX with the feedback of actual drug responses. But the MAE and MSE in both scenarios are very close, especially for the REP-RandomForest algorithm.

**Table 5:** Regression performance comparison using estimated and actual GEX values on Dataset1 and Dataset2.

	Method	Using estimated GEX		Using actual GEX	
		MAE	MSE	MAE	MSE
Dataset1	REP-ElasticNet	1.131	2.423	0.919	1.504
	REP-KNN	1.087	2.281	0.839	1.305
	REP-RandomForest	0.893	1.513	0.809	1.098
	REP-SVR	1.105	2.049	0.727	0.891
Dataset2	REP-ElasticNet	0.918	1.335	0.934	1.237
	REP-KNN	0.852	1.176	0.658	0.780
	REP-RandomForest	0.813	1.148	0.930	1.265
	REP-SVR	0.84	1.144	0.660	0.792

Columns corresponding to “estimated GEX” represent analysis in which in the test set, only the GEX values at the initial time point were used and the GEX of later time points were estimated. Columns corresponding to “actual GEX” represent analysis in which in the test set, GEX values of all time points were used.

## Conclusion

We studied the problem of drug response prediction for time-course gene expression data and presented a computational framework (REP) that: (1) has a recursive structure that integrates past drug response records for subsequent predictions, (2) offers higher prediction accuracy than several classical algorithms such as SVM and LR, (3) exploits the tensor structure of the data for missing GEX completion and unseen GEX prediction, (4) can predict drug response of different stages of a treatment from some initial GEX measurements. The achieved performance improvement for real data application suggests that our method serves as a better predictor of drug response using the time-course data.

## Supplementary information

Supplementary Information.
