# Supplementary material for: A recursive framework for predicting the time-course of drug sensitivity
Source: Sci Rep. 2020 Oct 19;10:17682. doi: 10.1038/s41598-020-74725-2 (PMC7573611; doi:10.1038/s41598-020-74725-2)
Supplement: Supplementary file 1 — Supplementary Information. [file 41598_2020_74725_MOESM1_ESM.pdf]

Supplementary of "A Recursive Framework for Predicting the Time-Course of Drug Sensitivity"

Cheng Qian[1], Amin Emad[2], Nicholas D. Sidiropoulos[1,*]

[1,*] Department of Electrical and Computer Engineering, University of Virginia, VA, USA

[2] Department of Electrical and Computer Engineering, McGill University, Montreal, QC, Canada

[*] corresponding. nikos@virginia.edu

Table S1 42 selected genes in Dataset-2

GZMB	ITGAL	IRF8	IRF5	GATA3	FAS	CD28	CIITA	ITGB2
IRF6	IRF3	JAK2	IRF2	IRF4	IFNAR2	JAK1	STAT3	CD86
NFKBIB	IL12RB2	CD69	MX1	HRAS	TRADD	MAP3K1	IFNAR1	CD22
GRB2	CD5	TYK2	IL10RB	IL12RB1	JUN	BAX	FOS	CD44
STAT4	STAT1	CD80	STAT2	ITGB1	NFKBIA			

Table S2 Regression performance comparison in terms of MSE at each time point using Dataset1.

Time (month)	3	6	9	12	18	24
REP-ElasticNet	**2.087**	**1.609**	**1.168**	**1.051**	**1.516**	**1.59**
Elastic Net	3.057	2.899	2.579	2.141	2.681	2.741
REP-KNN	**1.866**	**2.104**	**0.875**	**0.734**	**1.211**	**1.04**
KNN	3.938	3.477	3.547	2.443	2.701	3.31
REP-RandomForest	**1.655**	**1.142**	**0.483**	**1.178**	**0.967**	**1.164**
Random Forest	3.058	2.992	2.314	2.491	3.138	3.681
REP-SVR	**1.209**	**0.883**	**0.448**	**0.861**	**1.065**	**0.882**
SVR	3.407	3.051	3.324	2.618	3.062	3.2

Table S3 Regression performance comparison in terms of MAE at each time point using Dataset1.

Time (month)	3	6	9	12	18	24
REP-ElasticNet	**1.027**	**1.008**	**0.744**	**0.763**	**0.957**	**1.014**
ElasticNet	1.289	1.27	1.072	1.084	1.264	1.266
REP-KNN	**1.052**	**1.041**	**0.67**	**0.654**	**0.856**	**0.763**
KNN	1.513	1.326	1.409	1.13	1.269	1.422
REP-RandomForest	**0.931**	**0.877**	**0.53**	**0.854**	**0.782**	**0.881**

RandomForest	1.307	1.295	1.077	1.205	1.414	1.464
REP-SVR	**0.856**	**0.701**	**0.54**	**0.71**	**0.803**	**0.75**
SVR	1.357	1.228	1.255	1.129	1.344	1.376

Table S4 Regression performance comparison in terms of MSE at each time point using Dataset2.

Time	After 1 year	After 2 years
REP-ElasticNet	**1.202**	**1.272**
Elastic Net	1.418	1.476
REP-KNN	**0.822**	**0.738**
KNN	1.682	1.913
REP-RandomForest	**1.325**	**1.205**
Random Forest	1.588	1.917
REP-SVR	**0.798**	**0.786**
SVR	1.801	1.992

Table S5 Regression performance comparison in terms of MAE at each time point using Dataset2.

Time	After 1 year	After 2 years
REP-ElasticNet	**0.949**	**0.918**
Elastic Net	1.048	0.965
REP-KNN	**0.688**	**0.628**
KNN	1.144	1.114
REP-RandomForest	**1.004**	**0.855**
Random Forest	1.08	1.11
REP-SVR	**0.658**	**0.662**
SVR	1.191	1.101

Table S6 Regression performance comparison at each time point using Dataset1 and Dataset2. In Dataset1, there is only one patient has constant drug responses at different time points while the other 26 patients have time varying drug responses, i.e., there is at least one switch in the responses between two time points. In Dataset2, there are 14 patients with constant drug responses and 11 patients with time varying drug responses.

Drug response type	Dataset	Method	MSE	MAE	Method	MSE	MAE
Time Varying	Dataset1	REP-ElasticNet	**1.869**	**1.018**	ElasticNet	2.062	1.087
		REP-KNN	**1.844**	**0.987**	KNN	2.041	1.076
		REP-RandomForest	**1.270**	**0.840**	RandomForest	2.397	1.165
		REP-SVR	**1.854**	1.045	SVR	1.956	**0.994**
	Dataset2	REP-ElasticNet	**1.788**	**0.980**	ElasticNet	1.798	1.045
		REP-KNN	**1.647**	**0.980**	KNN	1.811	1.084
		REP-RandomForest	**1.527**	**0.908**	RandomForest	1.657	1.035
		REP-SVR	**1.388**	**0.862**	SVR	2.079	1.130
Constant	Dataset1	REP-ElasticNet	**16.833**	**4.070**	ElasticNet	18.841	4.341
		REP-KNN	**13.629**	**3.683**	KNN	16.558	4.058
		REP-RandomForest	**7.831**	**2.275**	RandomForest	14.548	3.662
		REP-SVR	**7.138**	**2.665**	SVR	19.128	4.373
	Dataset2	REP-ElasticNet	**0.980**	**0.870**	ElasticNet	1.172	0.977
		REP-KNN	**0.805**	**0.752**	KNN	1.232	0.998
		REP-RandomForest	**0.850**	**0.738**	RandomForest	1.372	1.043
		REP-SVR	**0.953**	**0.823**	SVR	**0.953**	0.833

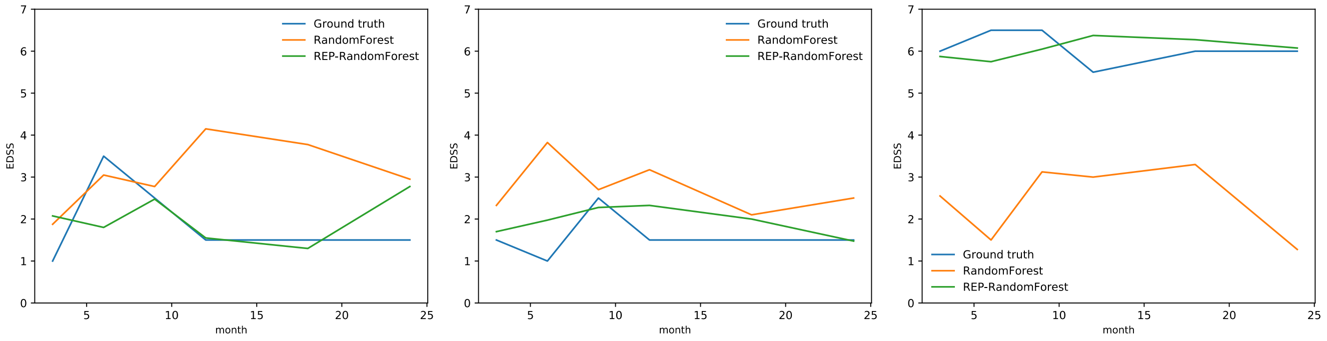

(1) REP-RandomForest versus Random Forest

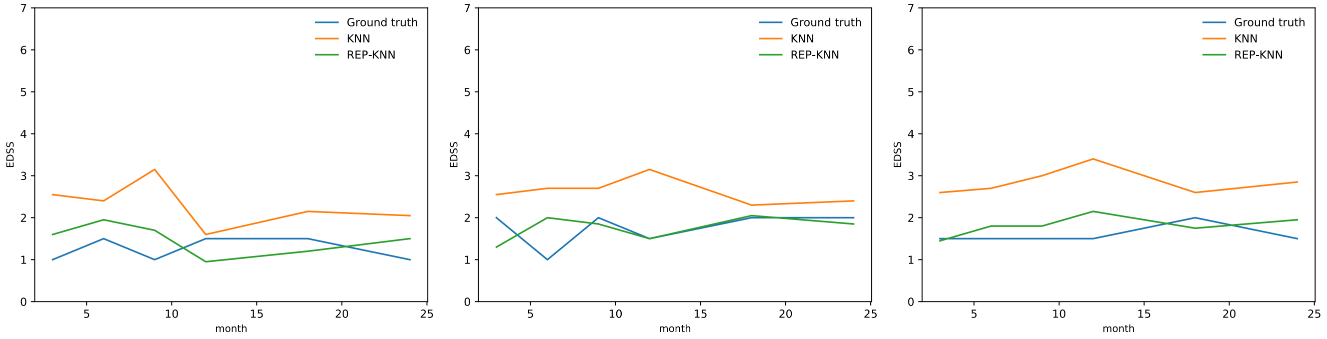

(2) REP-KNN versus KNN

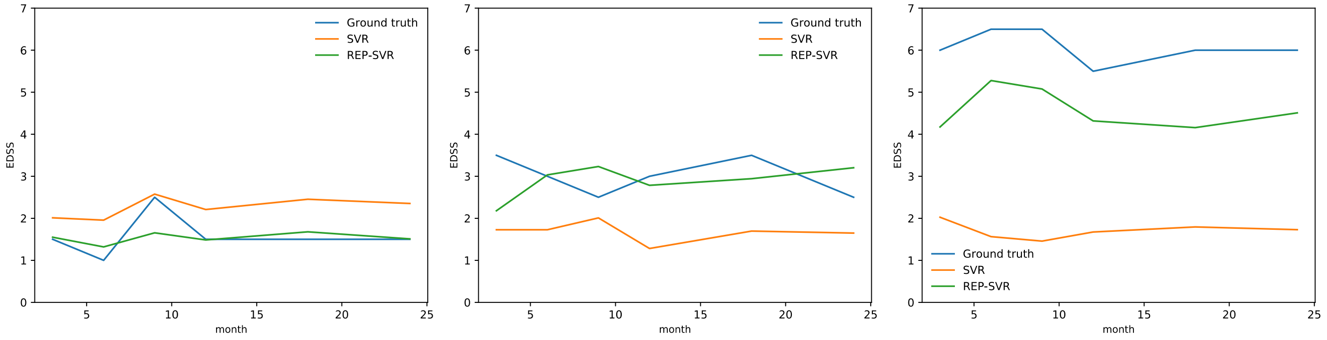

(3) REP-SVR versus SVR

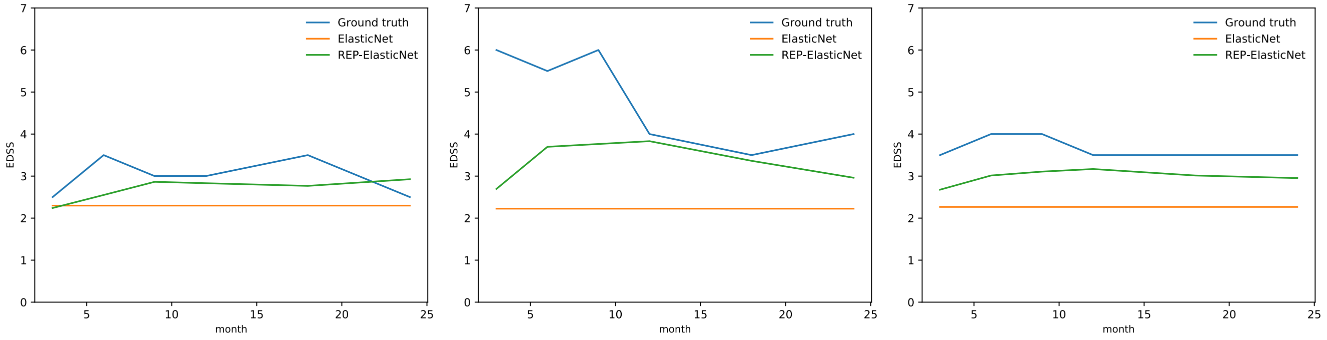

(4) REP-ElasticNet versus ElasticNet

Fig. S1 Examples of time-varying drug response prediction with and without the REP framework, where the ground truth EDSS values are from patients in Dataset1. Each subplot corresponding to a patient in Dataset1.

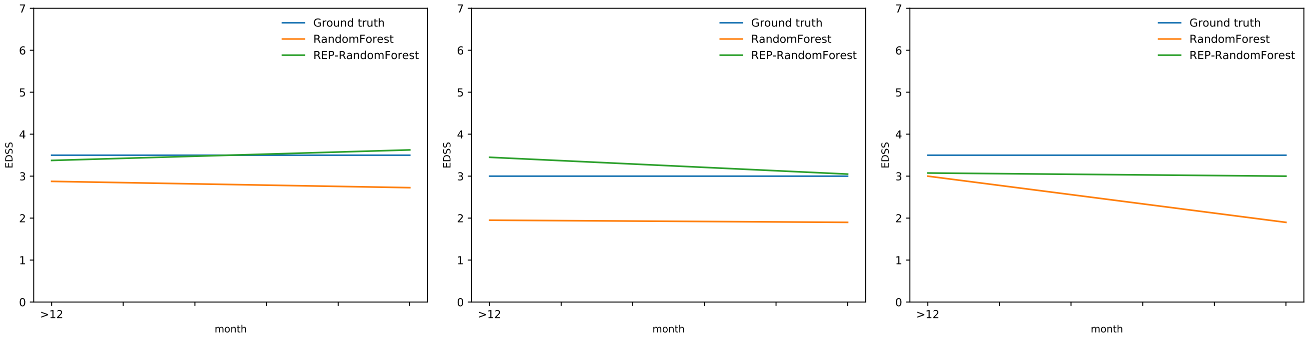

(1) REP-RandomForest versus Random Forest

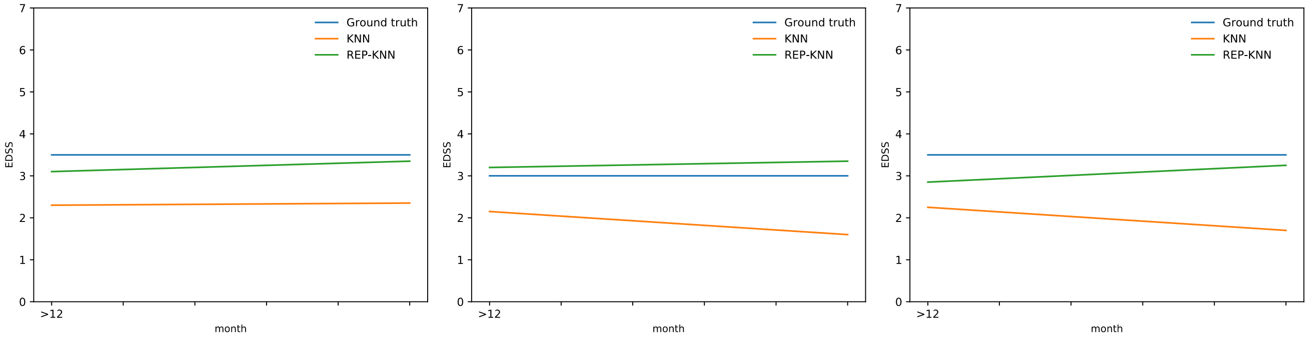

(2) REP-KNN versus KNN

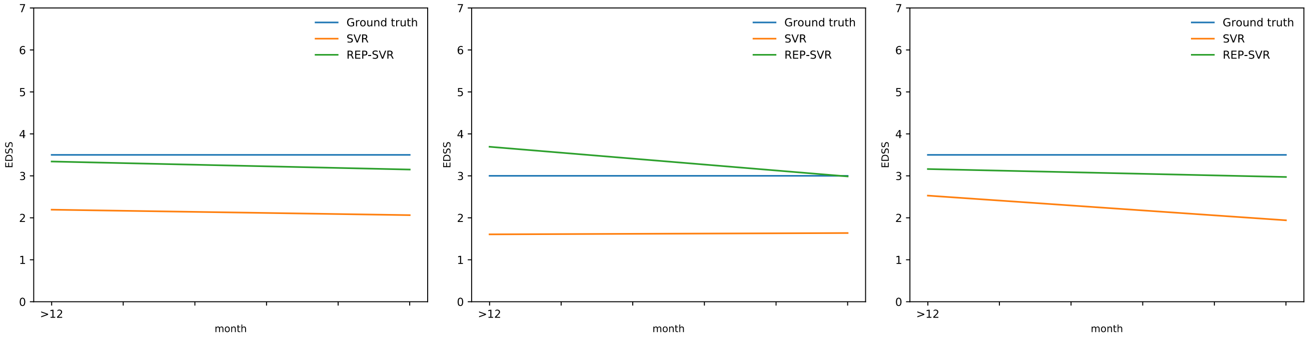

(3) REP-SVR versus SVR

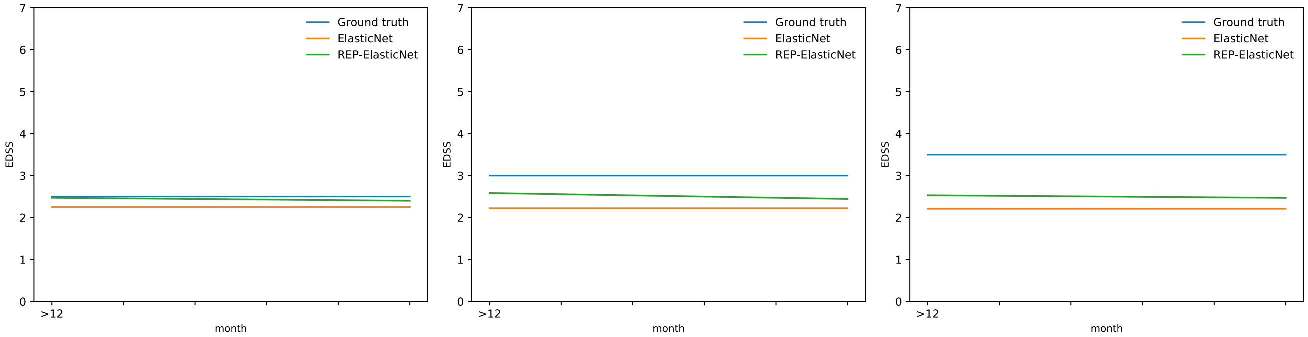

(4) REP-ElasticNet versus Elastic Net

Fig. S2 Example of constant drug response prediction with and without the REP framework, where the ground truth EDSS values are from the only patient in Dataset2 with constant drug responses. Each subplot corresponding to a patient in Dataset2.
